# Viscoelastic Behavior of Drug-Loaded Polyurethane

**DOI:** 10.3390/polym13162608

**Published:** 2021-08-05

**Authors:** Navideh Abbasnezhad, Mohammadali Shirinbayan, Fatiha Chabi, Stephane Champmartin, Abbas Tcharkhtchi, Farid Bakir

**Affiliations:** 1 Arts et Metiers Institute of Technology, CNAM, LIFSE, HESAM University, F-75013 Paris, France; mohammadali.shirinbayan@ensam.eu (M.S.); fatiha.chabi@gmail.com (F.C.); stephane.champmartin@ensam.eu (S.C.); farid.bakir@ensam.eu (F.B.); 2 Arts et Metiers Institute of Technology, CNAM, PIMM, HESAM University, F-75013 Paris, France; abbas.tcharkhtchi@ensam.eu

**Keywords:** drug-eluting stent (DES), polyurethane, mechanical properties, viscoelastic properties, drug delivery, flow rate

## Abstract

Drug-eluting stents are desirable platforms for local medicine delivery. However, the incorporation of drugs into polymers can influence the mechanical and physicochemical properties of said matrix, which is a topic that is still poorly understood. In fact, this is more noticeable since the apposition is most often accompanied by mechanical stresses on the polymer coating, which can induce therapeutic failure that can result in death. It is therefore necessary to better understand their behavior by examining their properties in conditions such as those in living beings. We studied polyurethane drug carriers made in-house. Diclofenac epolamine was chosen as a model hydrophilic medicine. We used thermal measurements (DMTA) and tensile tests. The aim was to establish the influence of the loading and release of the drug on the physicochemical properties of this polymer in the presence of a stagnant or circulating fluid medium, phosphate-buffered saline (PBS). For the two PU/drug loadings studied, the effect of the initial drug load was more marked. The free volume fraction and the number of pores in the samples increased with the increasing percent of the drug and with release time. The kinetic profiles were accelerated with the loading ratio and with the presence of flow. Young′s modulus and ultimate stress were not significantly influenced by the release time. A relevant relationship between the tensile properties and the viscoelastic behavior of the samples was developed. Our results have implications for optimizing the performance of drug coatings for stents.

## 1. Introduction

Polyurethane is the only type of polymer that can be in the categories of thermoplastics, elastomers, and thermosets. It is synthesized from the reaction between polyols and diisocyanates, wherein the choice of each polymer and the method of synthesis can drive it into different categories [1]. For this reason, PUs are used for various applications, such as adhesives, coatings, vehicle parts, sponge, implants and biological devices and organs [2].

In biomedical applications, PUs have also been used since they are biocompatible with effective control over mechanical strength and flexibility [3]. By monitoring the construction blocks and their final chemical composition, PUs may be made either biodegradable or bioinert. By regulating the structure of PUs, these properties can be adjusted according to the need. PUs can be classified as bioinert PUs and biodegradable PUs, the former of which are used as artificial organs in medical devices and as drug delivery carriers, such as drug-eluting stents (DESs).

Drug-eluting stents have altered therapeutic cardiology and resulted in a significant advancement in the prevention of coronary artery restenosis [4]. Nevertheless, their usage may lead to a delay in arterial wall healing, as well as endothelial function [5] and hypersensitivity responses produced by polymers [6], as well as an increased risk of late acute stent thrombosis. Second-generation DESs, which are more biocompatible and biodegradable polymers with different drug release formulations and designs, are related to a decrease in intra-stent restenosis (ISR) and, consequently, a decrease in the incidence of late stent thrombosis [7,8,9]. However, because of their permanent nature, they hinder the full recovery of vascular structure and function, raising the chance of stent failure very late [10]. Such fundamental restrictions with metal stents have encouraged the research community to innovate bioresorbable stent scaffolds, where they are intended to be temporary and disappear slowly, meanwhile providing vascular radial support. This is where the clinical reports showed more incidence of thrombosis after some years of implantation [11,12]. In a study by Wang et al. [13], they analyzed the failure of bioabsorbable scaffold stents (BRSs) and concluded that the polymeric microstructure from the initial steps of fabrication should be considered. They stated that even before chemical degradation, stress produced by crimping and inflation causes a loss of structural stability and variations in the polymer microstructure, where inhomogeneity of the microstructure and degradation of the polymer are the main reasons for this failure.

This issue is not just for the BRS, even the polymer used as a layer on the metal stent as a drug carrier should possess the critical physical, mechanical and chemical properties during release. Maintaining the proper physical and mechanical properties of the polymer both in the case of the coating used for stenting and as the stent scaffold itself has been very critical, where the ability to create a sustained release profile for drug release is part of an important strategy to examine drug-eluting stents [14].

In this regard, to improve the design and properties, studies have analyzed polymeric films [15,16], and the research has tried to improve the drug carrier′s properties and the viscoelastic behavior of the polymer [17,18,19,20,21]. In this area, researchers have always tried to obtain a more accurate estimate of the results by bringing the experimental environment closer to the natural environment [22,23].

According to studies, the importance of the flow rate to the stress created on stents [24,25] suggests that the presence of the flow rate creates a condition similar to the real condition. The role of the flow on the physical and mechanical properties of the material in this field of research has been emphasized.

Most polymer materials can possess the same necessary mechanical and non-toxic properties as biological materials [26,27]. Therefore, whether polymer materials can be used as medical materials generally depends on the biocompatibility of the materials themselves [28,29].

In this study, the physical and mechanical properties of pure PU films and those loaded with 10 and 20 wt% of a drug in static and dynamic conditions were investigated. The importance of the free volume fraction, the viscoelastic behavior and the tensile properties of PU films during the release time considering the two parameters of drug percentage and flow rate was analyzed.

## 2. Materials and Methods

### 2.1. Materials

Polyurethane was synthesized by the mixture of polyisocyanate (Raigidur VX) and polyol (Gyrothane 639) with the ratio of 2 to 5 according to the order in the notice. After completely blending the mixture, it was poured gently into the mold and then heated in the oven at a temperature of 50 °C for about 30 min to obtain the film of the polymer [25].

For the samples loaded with the drug, 10 or 20% (*w*/*w*) of the drug was added to the polyol, and then a complete mixture hardener was added to the solution. Later, the solution was cast in the mold covered with Teflon, followed by heating and drying. The rectangular films were cut in the dimensions of 2 × 5 × 30 mm^3^.

The drug used in this study was diclofenac epolamine (DE) as an example of a hydrophilic active substance, with a molecular weight of 318.13, and phosphate-buffered saline (PBS), which were purchased, respectively, from Genevrier Laboratory and Sigma Aldrich.

### 2.2. Characterization Methods

#### 2.2.1. Microscopic Observations

A scanning electronic microscope (SEM), HITACHI 4800, was used to qualitatively investigate the material microstructure and especially the evolution of the polymer morphology during the release test and fractography.

#### 2.2.2. Dynamic Thermo-Mechanical Analysis (DMTA)

For DMTA tests, the apparatus Dynamic Mechanical Analyzer type Q800 V21.2 was used. The dimensions of the rectangular specimens were approximately 30 × 5 × 2 mm^3^. The tests were performed over the entire temperature range of the apparatus at −70 to 0 °C with the step of 2.00 °C/min at multi-frequencies of 1, 2, 5, 10 and 30 Hz for calculation of the free volume fraction and at a constant amplitude of 1 Hz for the Cole–Cole equation. The storage and loss modulus were measured fitting to the temperature, and their corresponding values were calculated in this way.

#### 2.2.3. Mechanical Testing

Quasi-static tensile tests with a velocity of 5 mm/min were performed with an electromechanical and hydraulic system, an Instron 4301 machine, at the ambient temperature to see the effects of drug percentage, flow rate and the release on the mechanical behavior of the PU films. The time intervals chosen for the samples of the static and continuous state were 0, 1, 12, 24 and 96 h.

### 2.3. In Vitro Drug Release Procedure and Associated Measurement

**Figure 1:**
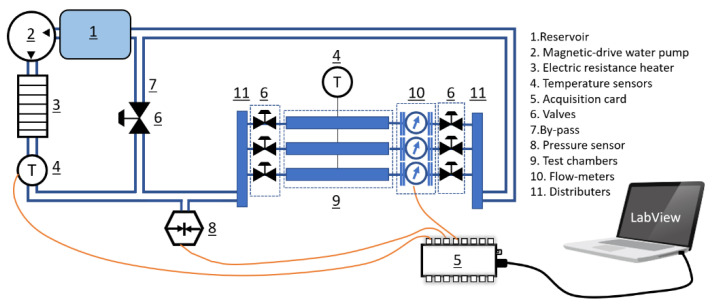
Schematic of the test bench.

In this regard, a test bench (Figure 1) was prepared to perform the tests on static and continuous states with different flow rates. At the static state, the prepared polymer films with the mass ratio of 10% of DE to PU were immersed in the closed chamber with 10 mL of phosphate-buffered saline (PBS) solution with a ratio of 60.0 mg/mL at 37 °C and pH 7.4. The release results were obtained at certain time intervals; at each time step, 4 mL was sampled for analyzing, and 4 mL of fresh PBS was added to the solution.

In the continuous state, the test bench could rotate the solution with the defined flow rate of 7.5 mL/s, which was controlled by a home-designed code in the LabView program. The sample was placed in the chamber and from one side it was in contact with the flow medium and the other side with a rigid surface. In order to avoid the moving of the sample by the flow, it was fixed with clamps. This test bench had a reservoir that contained 1 L of PBS, and, at each time step, 4 mL of the solution was sampled. The buffer solution was maintained at the temperature of 37 °C and the pH of 7.4. Figure 1 shows the schematic of the test bench.

The ultraviolet–visible (UV–Vis) spectrophotometer Lambda 35 was used to determine the concentration of diclofenac in the PBS solution. To calculate the accumulative release percentage of the drug, the total amount of diclofenac loaded to the PU was also measured after completely degrading the polymer film in the ultrasound. To establish the calibration curve of the measurements, the known concentrations of DE in the PBS were measured. The following calibration equation was found with the correlation coefficient of R^2^ = 0.999.

## 3. Results

### 3.1. The Effect of Flow Rate and Drug Percentage on the Drug Release

**Figure 2:**
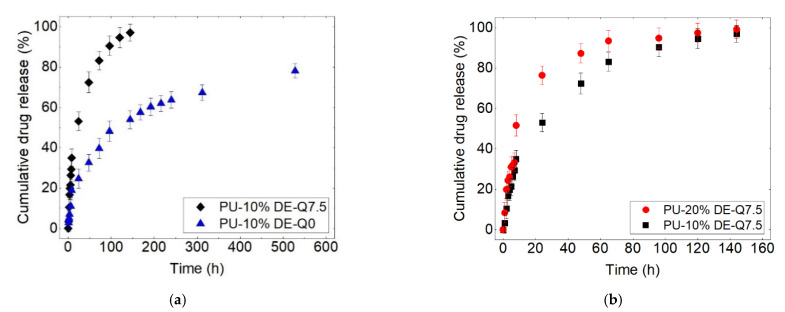
Cumulative drug release curves (**a**) at the flow rate of 0 and 7.5 mL/s and (**b**) PU-10%DE and PU-20%DE at the flow rate of 7.5 mL/s (PU-10%-DE-Q7.5: polyurethane-10% diclofenac epolamine—flow rate of 7.5 mL/s).

The objective of this study is to analyze the capacity of PU films as a drug delivery carrier and the evolution of polymer properties, such as physical, mechanical and viscoelastic behavior, considering the two different parameters of flow rate and drug percentage. At first, the results of the drug release in different conditions are presented. Figure 2 shows the drug release from the polyurethane samples at the static and continuous flow, with 10 and 20% of drug loading. The release profile for each case consists of two stages, respectively, burst release and extended release [30].

**Figure 3:**
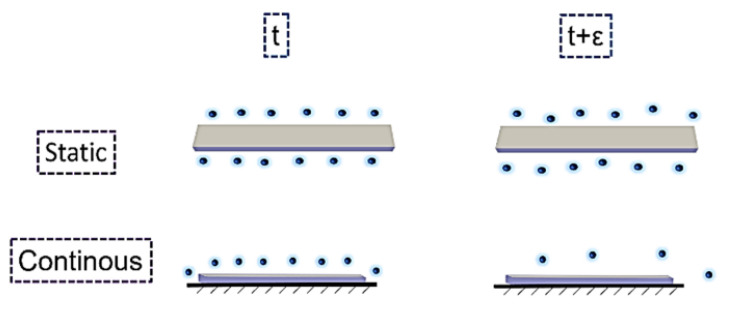
Gradient of concentration at t and t + ε for the static and continuous flow.

Figure 2a shows that increasing the flow rate decreases the overall time of the release. Moreover, it is notable that by changing the state of the flow from static to continuous, the amount of burst release is increased, which clearly shows that the burst phenomenon is related to convection. The second stage of the release, which is almost continued with the mechanism of diffusion, is slower for the flow rate of zero compared to 7.5 mL/s. This can be due to the high-water uptake by the polymer film when the flow rate increases. Moreover, in the static state, the drug is not able to migrate far away from the sample (thick mass boundary layer and low concentration gradients leading to low diffusive flux), whereas in the continuous state, the released drug is immediately transported by the flow (thin mass boundary layer and high concentration gradients leading to high diffusive flux). The former results reveal that the mechanism of diffusion is not irrelevant to the flow rate. This difference implies the effect of the gradient of concentration. One can consider the release at time t (shown in Figure 3) for the static and continuous states wherein the circulating medium, the gradient always stays high between the samples and the medium; however, in the static, although the medium is not saturated, the gradient will be low at time t + ε. One can note from the release curves that the same phenomena of release occur for the cases with different flow rates, where accelerating the flow increases the kinetics and speeds up the phenomena. It is notable that in the static state, the method was the incubation method, and two surfaces of the samples were in contact with the dissolution medium, but in the continuous state, just one side was in contact with the dissolution medium. Therefore, this emphasizes the importance of the flow rate on the release.

Figure 2b indicates that by increasing the initial drug load, the drug percentage released during the release test is higher, especially during the first stage, which is probably due to the presence of more drug particles on the surface, which results in the burst release of the drug. As mentioned in [31], the physicochemical properties of the active substance affect their distribution in the polymeric matrix, wherein it is more probable for the hydrophilic active substance to move toward the surface of the polymer. Therefore, the hydrophilic active substance that has more tendency to stay near the surface is more likely to result in burst release wherein the drug is released by desorption at the initial periods [31]. In addition, increasing the hydrophilic drug particles increases the water absorption, which is the requirement for the drug release from polymeric films. Therefore, increasing the drug percentage increases the rate of the release.

**Figure 4:**
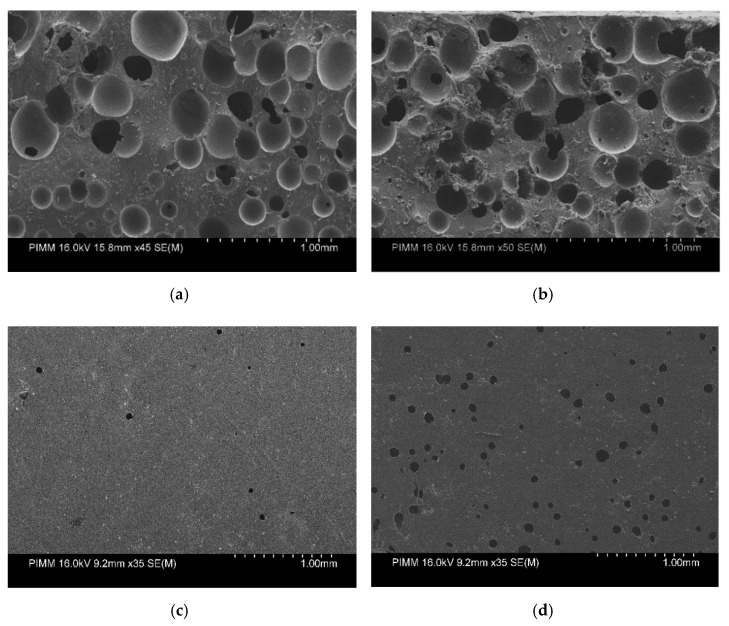
Micrographs of the (**a**) PU-10% DE and (**b**) PU-20% DE at Q7.5 after drug release of 96 h from the side and (**c**) PU-10%wt and (**d**) PU-20%wt from the surface side.

However, Figure 2b shows that at the second stage, the kinetics of the release decreases, and PU-10% and PU-20% are depleted from the drug at nearly the same time, but PU-10% comparatively pursues slower kinetics. It is observed from the release profiles that after the burst release, where the release is controlled by the diffusion phenomenon, the kinetics of the release decreases. This can be due to the higher initial burst release, which results in the gradient of the concentration decreasing and the slower kinetics of diffusion. The main reason for this can be explained by the scanning electron micrographs shown in Figure 4 from the side and surface of the samples, where increasing the drug percentage increases the pores in the structure of the samples. In addition, the possibility of the canalization between the pores contributes to the increased drug release.

Studies have concluded that apart from the external elements, the release behavior of the carriers is basically influenced by the polymeric structure and properties of the carriers. The subsequent results discuss these properties by considering the effect of two parameters, drug percentages and flow rate.

### 3.2. Polymer Properties Evolution during Drug Release

#### 3.2.1. Free Volume Fraction Evolution of PU

**Figure 5:**
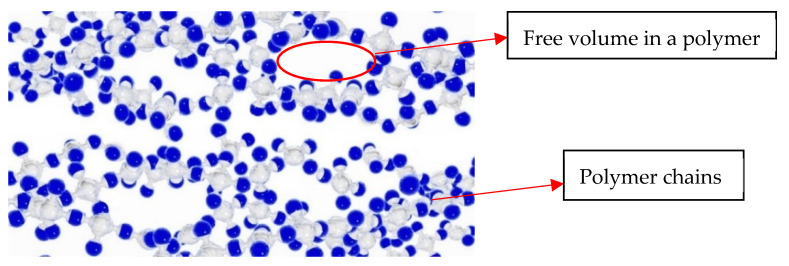
Schematic representation of the free volume in the polymers.

In a polymer, free volume can be determined by the volume of the total mass, which is not occupied by polymer chains and can therefore be a favorable place for the diffusing molecules, which can affect the permeability of the polymer membrane. Normally, the space or pores formed between the polymer chains are assumed to be the free volume fraction in the polymer. These free volumes are randomly distributed in the polymer, which reflects the movements of the polymer chains and, therefore, the transport of the molecules and, on the whole, the performance of the polymer membrane. It is difficult to analyze the pores directly in the membrane since the differences exist on a molecular scale. It is an inherent property that is temporary and variable, where the polymer′s physical state and density significantly affect its value. The mechanism of reducing the extra free volume of the membrane can be linked to physical aging, arising from the contraction of the lattice and the migration and diffusion of the free volume from the inside of the membrane to the surface [32]. These explanations reveal the importance of the polymer structure in the kinetics of the drug release. There are some studies that show the effect of the porous structure and the importance of the free volume fraction in drug release [33]. The polyurethane used in this study is an elastomer where in the structure small holes as the free volume are created. The effect of this parameter is investigated for the PU films without the drug and with 10 and 20% of the drug and its influence on the release behavior. Figure 5 represents a schematic of the free volume fraction in the polymers.

The Williams–Landel–Ferry (WLF) equation is one of the most used equations in polymer systems to calculate the free volume fraction in polymer membranes [34,35]. It is principally utilized to predict the mechanical properties of the material out of the time range of the experimental test.

The frequency of the applied loading affects the viscoelastic behavior of the polymer. This dependence between temperature and viscosity is proportional to the frequency and also the correlating relation. It describes the temperature dependence of the molecular relaxation times in the glass-forming substances at the glass transition temperature Tg [36]. The former is explained by the WLF equation:

(1) $$\mathrm{Log}\frac{f}{f_{r}}=-\frac{C_{1}\left(T-T_{r}\right)}{C_{2}+\left(T-T_{r}\right)}$$

where f is the frequency, T is the temperature, fr is the reference frequency (1 Hz) and Tr is the reference temperature.

(2) $$C_{1}=\frac{B}{f_{g}}{\;\mathrm{and}\;C}_{2}=\frac{f_{g}}{\Delta \alpha }$$

B is constant and near to 1, Δα is the thermal expansion coefficient and $f_{g}$ is the free volume fraction.

**Figure 6:**
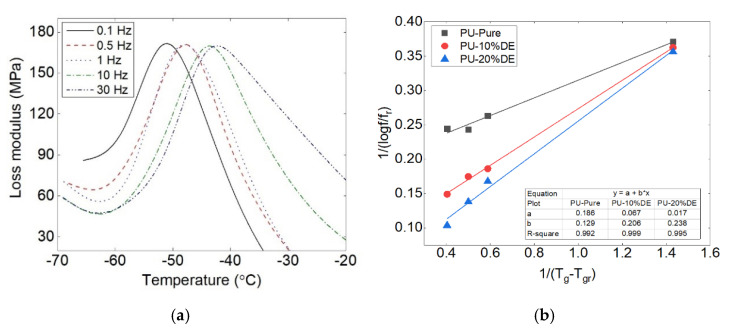
(**a**) Multi-frequency DMTA tests: glass transition temperature evolution (PU-10% DE) and (**b**) linear regression of WLF equation.

The linear regression approach is used to verify the validity of this equation, and $1/\log\left(f/f_{r}\right)$ is plotted versus $1/\left(T-T_{r}\right)$. If this plot is linear with $A\;={\;C}_{2}\;/{\;C}_{1}$ as the slope, then the WLF equation is validated. The increase in glass transition temperature during multi-frequency DMTA tests (Figure 6a) obeys the WLF theory. The linear regression coefficient was almost 1 (Figure 6b).

Therefore, the values of the free volume fraction coefficient using $f_{g}=\sqrt{\left(\left(B.\Delta \alpha .A\right)/2.303\right)}$ for PU, PU-10% DE and PU-20% DE are calculated, respectively, at about 0.37 × 10^−2^, 0.47 × 10^−2^ and 0.5 × 10^−2^.

It is notable from the results that the drug percentages change the free volume fraction of the polymer, as by increasing the drug percentage, the free volume fraction slightly increases. One can conclude that the effect of the drug concentration on the variation of polymer molecules in mixing should be considered, where it needs to be combined with the effect of the free volumes of the constituent solvent, polymer and drug in a polymer carrier film in determination of its thermodynamic properties.

#### 3.2.2. Mechanical Properties of PU Samples

**Figure 7:**
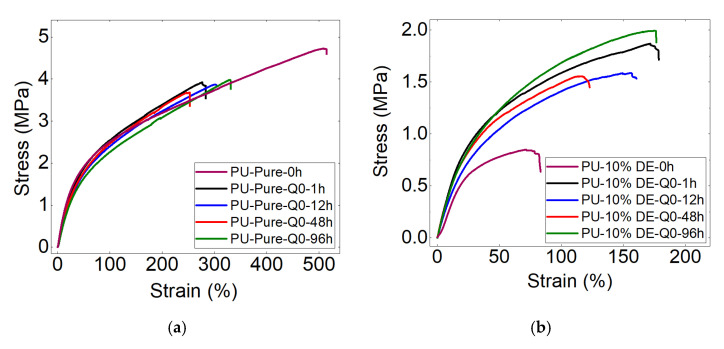

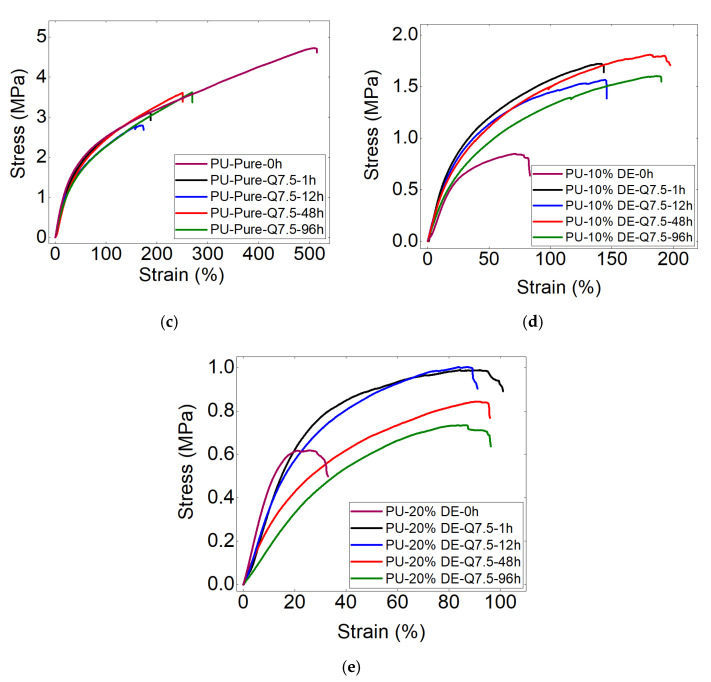
Stress–strain curves of (**a**,**c**) pure PU, (**b**,**d**) PU-10%DE and (**e**) PU-20%DE samples at different flow rates (PU-10%DE-Q7.5: polyurethane-10% diclofenac epolamine—flow rate of 7.5 mL/s).

Figure 7a,b shows the tensile curves of the PU samples with 0 and 10% of the drug at different times of incubation in the static state. The results show that the effects of the drug on the mechanical properties of the polymeric samples are significant. Figure 7a shows that the immersion time for the pure PU samples does not significantly change the tensile properties. However, Figure 7b indicates that increasing 10% of the drug decreased the mechanical properties to about half. Moreover, the presence of the drug causes the variation of the mechanical properties during the release time compared to the pure polymer. This can be due to the free volume and the pores created in the samples and the low bindings between the polymer chains due to the presence of the drug particles.

Figure 7c–e show the tensile behavior of the PU samples with 0, 10 and 20% of the drug at the flow rate of 7.5 mL/s. The results from these figures show that adding 10% of the drug decreases the mechanical properties to about half and adding 20% of the drug decreases them to about a quarter of the pure PU values. One can note from comparisons between Figure 7a,c as well as between Figure 7b,d that the flow rate of 0 and 7.5 mL/s for PU-Pure, stress and strain drops from 3.95 to 3.36 MPa and from 330 to 269, which shows that the effect of the flow rate on the mechanical behavior is not highly noticeable for this kind of polymer. Additionally, the mechanical properties of the PU samples with 0, 10 and 20% of the active substance before positioning in the fluid show the high effect of the drug load in the properties of the carriers (shown in Figure 7c–e). This indicates that adding the active substance results in low elasticity of the polymer carrier. After the initial contact of the drug-charged samples with the aquas medium (1 h) and the water absorption of the active substance and the polymer, the carrier becomes more deformable (shown in Figure 7d,e).

**Figure 8:**
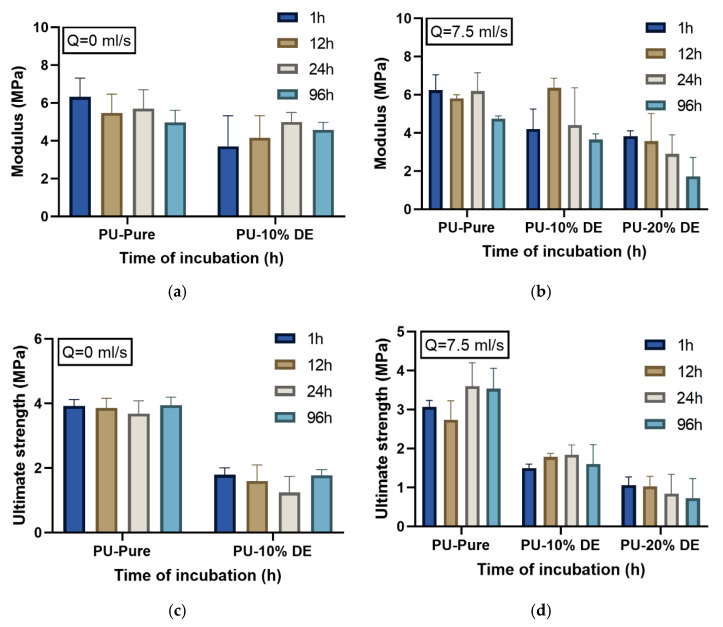

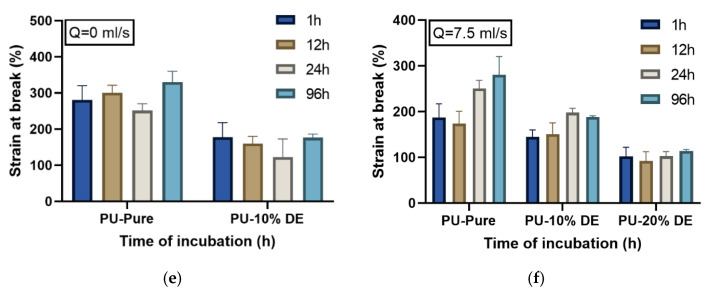
Tensile properties: (**a**,**b**) modulus, (**c**,**d**) ultimate strength and (**e**,**f**) strain at break of PU samples with different percentages of DE after release test in the static and continuous states.

Figure 8a–f show the comparison of the modulus, the ultimate strength and strain at break values for the different percentages of the drug (0, 10 and 20%) at different time intervals of the static and continuous release tests. The results from Figure 8a,b show that the effect of the drug percentage on the mechanical behavior of the samples is more significant in comparison to the flow rate, where the increase in drug percentage decreases the Young modulus value. The results show that the time of incubation slightly decreases the value of the modulus, where this effect is more visible for the charged polymers than for the pure polymers. Figure 8c,d show the ultimate strength of the PU with different percentages of the drug at the flow rates of 0 and 7.5 mL/s. By comparing these values, the impact of the flow rate and the time of incubation is not significant; however, increasing the initial load of the drug greatly decreases the ultimate strength of the PU samples. The variance in strength during the initial incubation time can be due to the plasticizing effect of the water molecules on the components of the DDS. Figure 8e,f show the strain at break for the PU with a different percentage of the drug at the flow rates of 0 and 7.5, respectively. Comparing the results of the strain at break for the PU shows that increasing the drug percentage decreases the elongation of the polymeric samples. However, elongation of the samples along with the incubation time does not differ significantly, but, because of the plasticizing after 96 h, it has a slight increase. From the mechanical results, one can observe that the highest modulus, stress and elongation at break among all the results with/without the drug before or after the test refers to the virgin pure PU. PU-20%DE before the incubation test has the lowest value of the stress and strain. This can be explained because as the percentages of the drug increase, the number of pores in the samples increases, which results in the decrease in the mechanical properties, whereas the flow rate does not remarkably affect the mechanical properties of the samples. The results show that by increasing the flow rate, the kinetics of the drug release is increased; however, this parameter does not significantly change the intrinsic properties of the material, such as the T_g_ and modulus, where the drug percentages change the mechanical properties of the polymer and increase the free volume fraction in the samples.

From the mechanical results, one can observe the higher modulus, strength and strain failure are related to the samples without the drug, where increasing the drug percentages decreases all mechanical properties of this type of polymeric material. This can be explained by the SEM figures, because as the percentage of the drug increases, the number of pores in the samples increases, which results in the decrease in the mechanical properties, whereas the flow rate does not affect the mechanical properties of the samples.

#### 3.2.3. Modeling of the Viscoelastic Behavior by the Cole–Cole Principle

**Figure 9:**
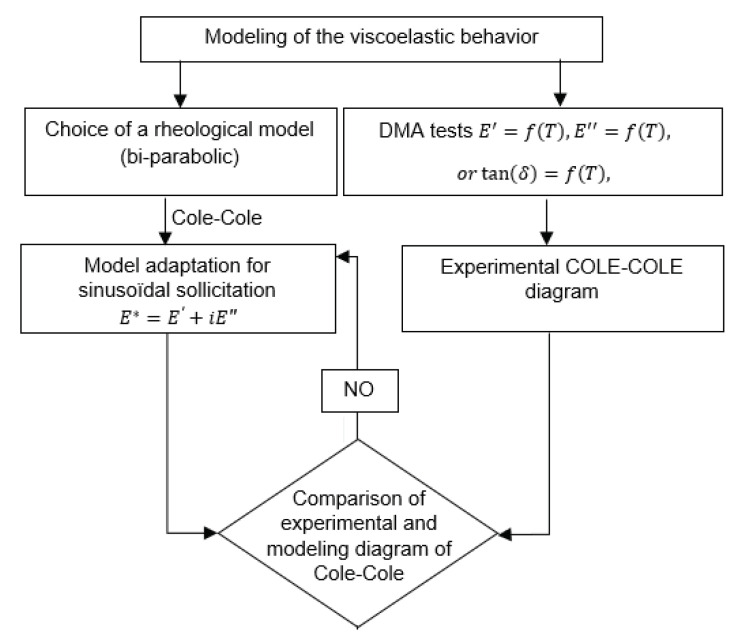
Flowchart diagram indicating the different steps of modeling $(E^{*}$: complex modulus, $E^{\prime \prime }$: loss modulus, $E^{\prime }$: storage modulus, δ phase lag between stress and strain).

Various approaches have been used to study the viscoelastic properties in the temperature range between the glassy and rubbery domain, and different models have been proposed to predict these properties. These models generally represent the curve of $E^{\prime \prime }$ (loss modulus) as a function of $E^{\prime }$ (storage modulus), and the curve is known as the Cole–Cole diagram [37]. Moreover, it describes the relaxation times and the temperature effect on viscosities. It is used to describe the dielectric relaxation of a material, which refers to the relaxation of a polymer when applying an external load. The changes in frequencies affect the viscoelastic properties because it has a direct effect on molecular architecture. In fact, when applying an oscillatory load, the macromolecule movements in the free volume present in the material system cause new overlaps with neighboring chains. This transformation carries out a significant role in the time of the relaxation phenomenon, which increases with the frequency. The diagram below (Figure 9) illustrates the different steps of this work.

The polarization magnitude of a material is defined by the dielectric constant represented by the equations of Debye and Onsager [38]. The single relaxation peak is inappropriate to reasonably describe the viscoelastic behavior of polymers. Cole–Cole is an appropriate treatment of dielectric relaxation data marked by plotting $E^{\prime \prime }$ versus $E^{\prime }$ [39]; each point corresponds to one frequency [40]. Structural changes occurring in cross-linked polymer materials can be carried out using the Cole–Cole principle.

**Figure 10:**
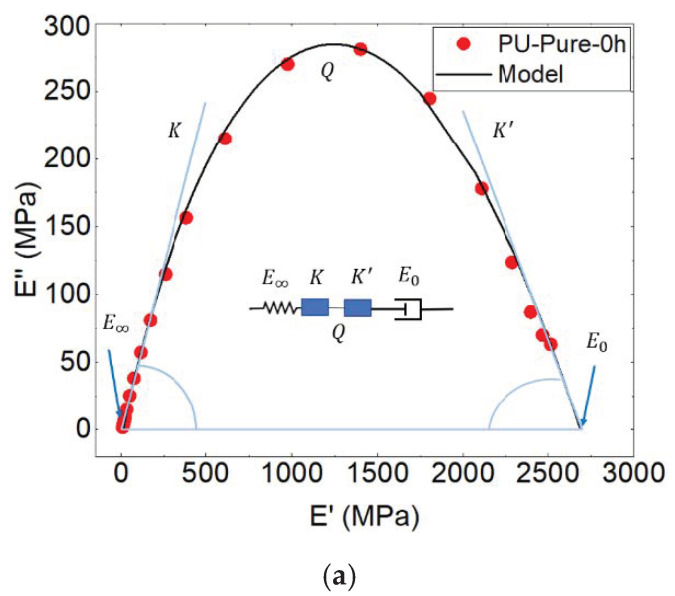

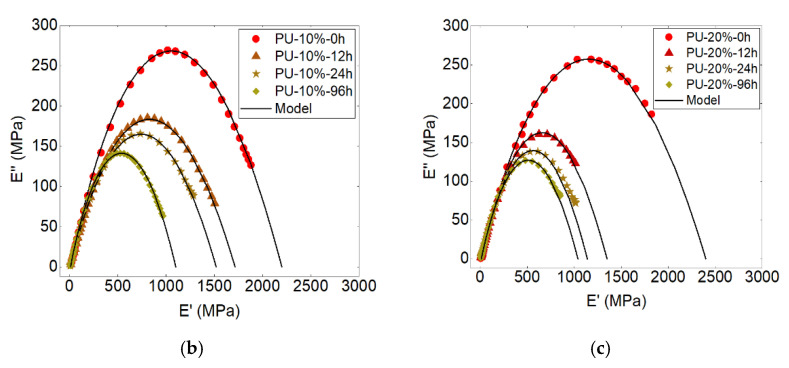
Cole–Cole plot of polyurethane (**a**) PU-Pure, (**b**) PU-10% and (**c**) PU-20% of drug at different times of release at the flow rate of 7.5 mL/s (f = 1 Hz).

For the validation of the theoretical model, experimental data obtained by the DMTA tests were required. After these tests, an asymmetric Cole–Cole diagram was plotted (Figure 10).

According to the Perez model [41,42] the behavior of polymers can be explained by the bi-parabolic model presented by the following equation:

(3) $$E^{\prime }=E_{\infty }+\left(E_{0}-E_{\infty }\right)\frac{1+\cos(\frac{k\pi }{2})\left(\omega \tau {)}^{-K}+Q\cos(\frac{k^{\prime }\pi }{2}\right){\left(\omega \tau \right)}^{-K^{\prime }}}{D}\;$$

(4) $$E^{\prime \prime }=\left(E_{0}-E_{\infty }\right)\frac{\sin(\frac{k\pi }{2})\left(\omega \tau {)}^{-K}+Q\sin(\frac{k^{\prime }\pi }{2}\right){\left(\omega \tau \right)}^{-K^{\prime }}}{D}\;$$

and

(5) $$D=\left[1+\cos(\frac{k\pi }{2}\right)\left(\omega \tau {)}^{-K}+Q\cos(\frac{\;k^{\prime }\pi }{2}\right){\left(\omega \tau {)}^{-\;K^{\prime }}\right]}^{2}+\left[\sin(\frac{k\pi }{2}\right)\left(\omega \tau {)}^{-K}+Q\sin(\frac{\;k^{\prime }\pi }{2}\right){\left(\omega \tau {)}^{-\;K^{\prime }}\right]}^{2}$$

where k, $k^{\prime }$ and Q are the constants of this model. ω =2πf is the angular frequency (f = frequency). Furthermore, $E^{*}$ is the complex shear modulus: $E_{\infty }$ and $E_{0}$ are the value of the modulus at the, respectively, glassy and rubbery states. k and $k^{\prime }$ depend on the slope of the tangents at the beginning and the end of the Cole–Cole diagram (${\mathrm{dE}}^{\prime \prime }/{\mathrm{dE}}^{\prime }),$ and Q is a constant related to the maximum value of $E^{\prime \prime }$. τ is the average relaxation time.

To investigate the viscoelastic behavior, the Perez model was applied in this study using the below equation:

(6) $$E^{*}=E^{\prime }+{\mathrm{iE}}^{\prime \prime }=E_{\infty }+\frac{E_{0}-E_{\infty }}{1+{\left(i\omega \tau \right)}^{\kappa }+Q{\left(i\omega \tau \right)}^{\kappa^{\prime }}}\;$$

where $E_{\infty }$ is the shear modulus corresponding to the rubbery state, and $E_{0}$ corresponds to the modulus at the glassy state. Each transition is described by a pair of ($E_{0},E_{\infty }).$ k and $k^{\prime }$ are identified with the slope of (${\mathrm{dE}}^{\prime \prime }/dE^{\prime }),$ from both sides of the curve. k indicates the intensity of the correlation effects occurring during the expansion of the molecules (changes in shape, area and volume) and the molecular motion; a rise in this value indicates decreased molecular mobility [43]. $k^{\prime }$ describes the difficulty with which local shear is developed. The mobility of polymer chains becomes slower as $k^{\prime }$ decreases [44]. The parameter Q is a function of the concentration of quasi-punctual defects, and it is related to the maximum value of $E^{\prime \prime }$, which is higher when Q is lower. The parameter τ is related to the chain relaxation time, describing the time taken by the structural unit to move at a distance comparable to that of its dimensions to return to its thermodynamic equilibrium. Indeed, the molecular mobility decreases with the temperature, and this decrease corresponds to the decrease in thermal activation but also to the increasingly marked cohesion when the temperature decreases (decrease in enthalpy, entropy of configuration and specific volume); the rearrangement time becomes very long compared to these local movements. The values of the model′s parameters are shown in Table 1.

The theoretical curve fits perfectly with the experimental results (Figure 10), signifying that the bi-parabolic model can accurately predict the viscoelastic behavior of PU. One can note that the viscoelastic behavior of PU (with 0, 10 and 20% of DE) obeys the Perez model. This confirms the importance of the matrix in the behavior of PU with and without the drug.

One can note from Table 1 that the elastic modulus for the pure PU is higher; however, by increasing the drug percentage and the release time, the elasticity of the samples decreases. The same logic is shown for the viscous modulus wherein the pure PU is higher, and by increasing the drug percentage and release time, it decreases.

Moreover, analysis of the results obtained shows that by increasing the drug percentage, the value of k decreases, which indicates the faster motion of the molecules.

Furthermore, k and $k^{\prime }$ decreases with the increasing viscosity and compactness of the samples; therefore, it is observed from Table 1 that the samples of PU-20% at the times 0, 12 and 24 h have the same value, and this value increases when the time of the release is increased to 96 h. One can observe that by increasing the drug percentage and the release time, the relaxation time increases.

**Figure 11:**
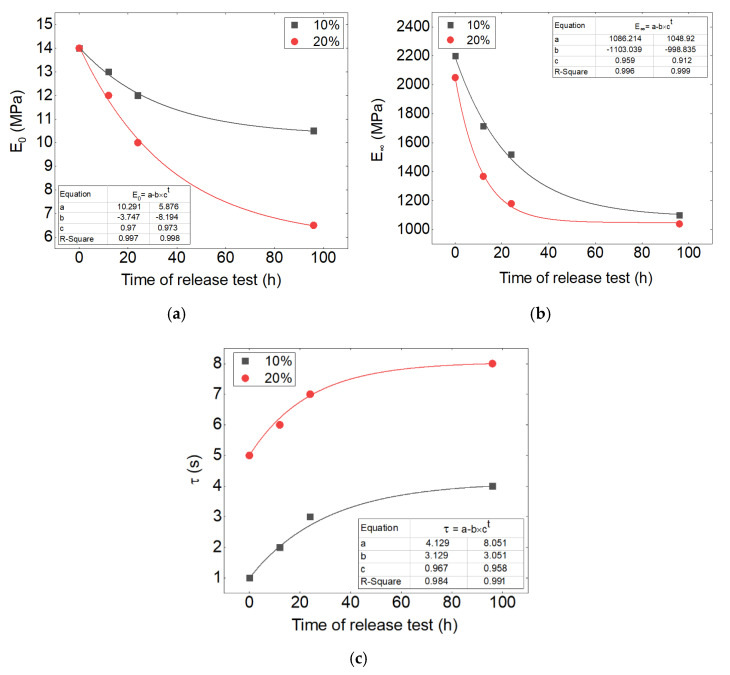
Relationship between the values of (**a**) elastic and (**b**) viscous modulus and (**c**) relaxation time with the time of release and drug percentage.

The evolution of storage and loss moduli and the relaxation time at different times of release can be analyzed for each drug percentage. Figure 11 shows these curves. One can observe from Figure 11a,b the decrease in viscous and elastic moduli. The relationship of the release time and these parameters shows an exponential equation type$\;y=a-b\times c^{t}$. The latter is observable for both 10 and 20% of the initial drug loaded. The effect of the initial drug load on the equation of the viscous modulus has a double increase on the parameters of “a” and “b” when increasing the drug from 10 to 20%. The former shows that there is a direct relationship between these parameters and the drug percentage. However, as presented in Table 2, the variables of the equation for the elastic modulus do not change remarkably for neither 10 nor 20%, where it indicates less dependence of the elastic modulus on the drug percentage.

Moreover, the investigation about the relaxation time shows that relaxation time is increased during the release time. The effect of the initial drug load on the equation of the relaxation time shows a double increase on the parameter “a” of the exponential equation.

This analysis can be used to predict the viscoelastic behavior of the samples with different drug percentages during the release phenomenon.

## 4. Conclusions

Understanding the influence of the parameters of a drug on the behavior of the carrier polymer matrix and vice versa is of major interest, particularly in the field of drug-eluting stents. In this study, PU/drug samples were investigated in vitro regarding the influence of the drug loading ratio and the presence or not of PBS circulation on the thermal and mechanical properties of the polymer matrix. Diclofenac epolamine was blended with PU in ratios of 0, 10 and 20 *w*/*w*% with respect to the polymer. Our results showed that by increasing the flow rate, the kinetics of the drug release increased; however, this parameter did not significantly change the intrinsic properties of the material, such as the mechanical properties. In contrast, an increase in the drug percentage increases the kinetics of the release, changes the tensile properties of the polymer and increases the free volume fraction in the samples. Thus, the higher mechanical properties refer to the polyurethane without the drug, whereas the lower mechanical properties refer to the loaded polyurethane samples before the drug liberation. The viscoelastic properties of the samples were additionally affected by increasing the drug percentage and the release time; the elastic and viscous modulus decreases whereas relaxation time increases. It is notable that these characteristics follow an exponential relationship with time release.

## Figures and Tables

**Table 1:** Perez model parameters for PU and PU-10% and PU-20% of diclofenac at different release times at the flow rate of 7.5 mL/s (f = 1 Hz).

Samples	$E_{\infty }$ (MPa)	$E_{0}$ (MPa)	k	$k^{\prime }$	Q	τ (s)
PU-Pure-0 h	16	2690	0.41	0.23	2	0.5
PU-10%-0 h	14	2200	0.4	0.25	1.2	1
PU-10%-12 h	13	1715	0.37	0.24	2	2
PU-10%-24 h	12	1519	0.37	0.25	2.4	3
PU-10%-96 h	11	1100	0.45	0.3	3	4
PU-20%-0 h	14	2050	0.24	0.3	2	5
PU-20%-12 h	12	1368	0.24	0.34	2	6
PU-20%-24 h	10	1180	0.24	0.34	2	7
PU-20%-96 h	7	1040	0.25	0.35	2	8

**Table 2:** Parameters of the relationship between the values of elastic and viscous modulus and relaxation time with the time of release and drug percentage.

Drug Percentage	Parameters of Equation	a	b	c	R^2^
10%	$E_{0}$ (MPa)	10.291	−3.747	0.97	0.997
	$E_{\infty }$(MPa)	1086.214	1103.039	0.959	0.996
	τ (s)	4.129	3.129	0.967	0.984
20%	$E_{0}$ (MPa)	5.876	−8.194	0.973	0.998
	$E_{\infty }$(MPa)	1048.920	998.835	0.912	0.999
	τ (s)	8.051	3.051	0.958	0.991

## Data Availability

The data presented in this study are available on request from the corresponding author.
